# Reducing Repeat Emergency Department Visits for Low-Acuity Patients Using a Healthcare Connection Program

**DOI:** 10.5811/westjem.25357

**Published:** 2025-06-25

**Authors:** Mitchell Hoyer, Kimberly A. Stanford, Ernestina Perez, Rachel Nordgren, Laura Markin, Melanie Francia, Zain Abid, Marika Kachman, Brenda Battle, Thomas Spiegel

**Affiliations:** * University of Chicago Medicine, Department of Urban Health, Chicago, Illinois; † University of Chicago Medicine, Department of Emergency Medicine, Chicago, Illinois; ‡ University of Chicago, Department of Biostatistics, Chicago, Illinois; § University of Chicago Medicine, Department of Biological Science, Chicago, Illinois

## Abstract

**Background:**

Emergency department (ED) utilization for non-emergent issues has been a longstanding issue in the United States, especially in service areas with high Medicaid enrollment. The Medical Home and Specialty Care Connection Program (MHSCC) at University of Chicago Medicine (UCM) supports patients recently seen in the ED with follow-up care by assisting patients with follow-up appointments, establishing a medical “home” and providing education on primary care utilization via working with a patient advocate. These types of programs have inconsistent results throughout the literature and a dearth of study periods. We conducted a program evaluation to assess the association of the MHSCC in reducing low-acuity ED utilization for program patients.

**Methods:**

This program evaluation used retrospective data from the MHSCC program dataset from 2012–2020 and matched with electronic health records of low-acuity ED visits at UCM ED from 2010–2022 for each patient. Pre- and post-low-acuity ED visit rates were calculated based on the patients first program enrollment and compared using the Wilcoxon signed-rank test.

**Results:**

In total 5,482 ED patients enrolled in the program were included in the sample, 537 of whom were enrolled more than once. These patients had 41,530 low-acuity ED visits. The rate of low-acuity ED visits after the program enrollment was significantly lower than before with a mean of 2.5 visits per year before program intervention to 1.38 after, a 45% decrease (*P*<.0001). This resulted in an estimated 9,487 fewer low acuity ED visits over nine years. Patients with multiple enrollments (up to four) further resulted in a slightly lower ED visit rates. Patients who benefitted the most in both proportion and mean analyses were of low acuity.

**Conclusion:**

We found a significant reduction in program patient’s ED visit rates for low-acuity needs. Further evaluation on the outcomes of the program, mechanisms of physician referrals and attributes of the patient population are recommended to understand what drives these findings.

## INTRODUCTION

Emergency department (ED) usage across the United States has a track record of increasing in number of patients through 2019, outpacing growth in population, and cost.^1^–^5^ From 2010–2016 there was a significant increase in both ED visits and mean charges for patients (129 million visits per year to 145 million, and $2,061 per visit to $3,516, respectively).^2^ Although, ED expenditure is a small proportion of overall health spending, it increased in amount and overall proportion during this same time period.^1^,^2^ Even with COVID-19 related ED use reductions through 2022, many patients still frequent the ED with low severity of illness or exacerbations preventable through consistent primary care.^4^,^6^–^9^ Furthermore, the high usage of EDs can lead to negative outcomes due to ED crowding and long wait times, which are both inconvenient for patients and can lead to patients leaving without being treated.^10^ Modern data surveillance and monitoring identifies potential drivers of this situation, as in 2016, 14% of Americans reported “not having a regular source of care” and “barriers to accessing primary care” as top reasons for visiting the ED, particularly among Medicaid enrollees.^11^–^14^ A frequent intervention of many hospital systems for reducing ED revisits is through patient assistance, education, and linkage to care programs.

At the University of Chicago Medical Center (UCMC) ED, this response is carried out by the Medical Home and Specialty Care Connection Program (MHSCC). As the flagship program of the Urban Health Initiative (UHI), the main program objectives are to assist patients with scheduling follow-up care appointments, establishing a medical “home,” and providing education on primary care utilization. These objectives are implemented by patient advocates. Patient advocates, who are trained similarly to community health workers, incorporate a broader focus on patient medical and social determinants of health (SDoH) support beyond the ED. Although existing literature has shown some similar programs have yielded beneficial results in meeting these objectives, the overall landscape has shown variable, inconsistent findings regarding statistically significant reductions in ED revisits with a few studies reporting increased ED revisits.^15^–^27^ As interest in these types of programs increases, it will be important to understand their impact on ED utilization.

Our goal in this study was to evaluate the impact of the MHSCC program on repeat, low-acuity ED visits, hypothesizing that patients who accepted help from a patient advocate through the MHSCC program would result in a reduction in their low-acuity ED visit rate.

## METHODS

### Program Details and Study Population

UCMC is a Level I trauma center that is part of a large, tertiary-care hospital system. It houses the largest ED on the South Side of Chicago, a community affected by many SDoH. 28 The MHSCC program stationed within this system identified patients through various means. Originally, patients were screened by patient advocates based on acuity level and need for a primary care physician. Starting in 2014, a paper-based physician referral pathway was created for those times when patient advocates were not staffed (nights, holidays, weekends, etc). This transitioned into the electronic health record (EHR) via a physician order in 2018 (comparable to any other consultation request) without any strict inclusion criteria.

**Population Health Research Capsule**

What do we already know about this issue?

*ED use for non-emergent concerns continues to be an issue in the US. Medical home connection programs have inconsistent results throughout the literature*.

What was the research question?

*Was there a reduction in low-acuity ED utilization for program patients from before and after their program intervention?*

What was the major finding of the study?

*Low-acuity ED visit rates were lower after the program with a mean of 2.5 visits/year before to 1.38 after, a 45% decrease, P<.0001*.

How does this improve population health?

*Implementing these program models in similar settings could improve local health care connection and reduce reliance on the ED for low-acuity needs*.

This EHR system change likely led to a substantial increase in physician referrals and overall demand for the program during the study period, respectively, and eventually eliminated ad hoc screening by patient advocates. As the team size did not grow during the study period, it is probable there were patients who were not outreached to due to program capacity. Patients were approached by patient advocates for program enrollment both in person during their ED visit and over the phone after being discharged from the ED. The intervention consisted of providing assistance with scheduling primary and specialty care appointments, healthcare access resources and healthcare navigation education. Follow-up phone calls were often conducted two days before the patient’s scheduled appointment as a courtesy reminder. Most work occurred within one week of a patient’s discharge from the ED, and patient advocates did not follow patients for an extended period. Because of the brevity of the program, program enrollment, participation, and intervention are used interchangeably depending on context.

In this retrospective cohort study we used data collected in the MHSCC program activity database (MHSCC dataset) from January 2, 2014–July 24, 2020 to track program operations. The data includes variables on patient index visits (ED visit when program enrollment took place), support and intervention provided, and other information on the patient and program enrollment. From this database, the study sample included adult ED patients during this period. Several other inclusion criteria were selected to further define the scope of the study. For some patients, it was unclear whether the program enrollment was associated with an adult ED visit because the time between the last ED visit and enrollment was greater than 30 days. We excluded those cases from the study. Additionally, an extensive literature review showed that similar programs had mixed results regarding the effectiveness of these interventions for high-frequency ED users and, thus, those users were removed from our sample dataset a priori.^9^,^22^ We defined high-frequency ED use as patients with more than 21 visits over the study period (above the 95^th^ percentile of the sample). Although the ED in question is an adult ED, patients as young as 16 are treated under certain circumstances, and this study included 18 patients ages 16–17 in the analysis. Lastly, patients without a valid medical record number and those who were deceased within the study period were removed from the dataset.

The MHSCC dataset was merged with data from our EHR (Epic Systems Corporation, Verona, WI) from January 1, 2012–December 31, 2022. Data from the EHR included all patients’ ED visits, demographic information and other relevant data such as ED visit acuity levels, and dates of each respective ED visit. Index ED visit dates were then matched to patients based on the program enrollment date. We included prior and subsequent ED visits from the EHR data only if they were recorded as low acuity (Emergency Severity Index level 4 or 5).^29^ We included all low-acuity ED visits two years before and two years after the MHSCC dataset included in the merged dataset to evaluate ED visit rates for patients enrolled at the beginning and end of the MHSCC dataset. All index visits were included regardless of acuity level.

When reviewing medical records for the study, we followed several criteria for retrospective chart review best practices: abstractor training; aforementioned case selection criteria; variable definitions; monitoring abstractors’ work; interobserver reliability discussions; and sampling methodology for program participants.^30^ Furthermore, only patients with complete data were used in the statistical analysis. Approval for this study was obtained from an institutional review board, which deemed the study exempt with no stipulations and consent waived.

Overall, the study was outlined by analyzing three parameters: 1) effectiveness of the program in reducing low-acuity ED use; 2) effectiveness of multiple program enrollments for a patient; and 3) description of patient characteristics associated with the success of this intervention.

### Data Analysis

We reviewed and compared study population descriptive statistics between groups respective to both main and sub-analyses. Demographic and medical information analyzed included age, sex, race, ethnicity, insurance status (payor group), and number of low-acuity ED visits. Demographic frequencies were compared with proportions, testing for significance using chi-square test for categorical variables (the Yates correction for continuity was used for tables with a value of zero), while means were compared using *t*-test and analysis of variance. For patients with multiple enrollments, the demographics during their first index visit were used in descriptive tables. Although not part of the analysis methodology, we evaluated both the MHSCC dataset and EHR data to develop a control group. Alpha for significant values was set at .001 due to the large sample size. Data cleaning occurred in R 4.2.2 (R Foundation for Statistical Computing, Vienna, Austria) and Microsoft Excel 2016 (Microsoft Corporation, Redmond, WA), and statistical analyses and graphs were done in R (4.2.2).

### Main Analysis

For the primary analysis, we made a comparison between a patient’s low-acuity ED visit rates before and after program enrollment. This included calculating the rate of low-acuity visits before the program intervention (pre) and the expected rate of low-acuity ED visits afterward (post) (adjusted for time after the enrollment). We calculated the post rate by taking the observed ED visit rate before program intervention and multiplying it by the number of months afterward in two methods: 1) 12 months post; and 2) until the end date of study period (December 31, 2022). The rate of observed low-acuity ED revisits was then compared to the expected value using the Wilcoxon signed-rank est.

Further comparison of different demographics characteristics were made on the outcome of interest based on whether the patient had any reduced visit rate using bivariate analysis (“reduced low-acuity ED visit rate” vs “no reduced low-acuity ED visit rate”). These comparisons were also done with separating out patients who had a “near zero” difference in their ED visit rate. The “near zero” group included any patients having a change in low-acuity ED visit rate between −0.5 and 0.5 visits per 12 months when looking at the entire study period. We calculated mean low-acuity ED visit rate changes post-program intervention for demographic categories that demonstrated significant differences.

### Subanalysis

The subanalysis investigated whether multiple program enrollments with a patient advocate during the evaluation period further reduced the rate of low-acuity ED visits. This was analyzed with multiple tests of the Wilcoxon signed rank test among subgroups of patients: those with one enrollment; and those with two or more enrollments. We compared demographic and frequency data for patients with one and two or more program enrollments. Both main and subanalyses were used to estimate the number of ED visits prevented over the course of the study. This was done by summing the individual differences in observed vs expected low-acuity ED visits.

### Program Healthcare Cost Prevention

To assess the impact of the MHSCC program on healthcare costs, we performed an analysis of the average total variable cost for program patients. Patients who were referred to the MHSCC program from July 2021– June 2022 with low-acuity ED visits were used to calculate an average direct variable cost. This was calculated by internal hospital finance teams. This average was then multiplied by the estimated total number of low-acuity ED visits prevented for each year in the study. Direct variable costs are those accrued during the patient’s ED visit and treatment excluding fixed costs such as labor (nurse, physician salary, etc).

## RESULTS

From 2014–2020, 50,206 patients were enrolled by patient advocates. Of these patients, 9,626 were eligible for the study, of whom 5,482 (57%) participated in program interventions and 4,144 (43%) declined assistance. The 5,482 eligible patients who accepted patient advocate services were mainly Black (95%) and female (62%) with a mean age of 38.4 (SD 16.2) years. The largest proportion of index visits were designated as acuity level 4 (56%), followed by level 3 (30%). Payor groups for patients included private insurance (17%), Medicaid (54%), Medicare (9%), uninsured (13%), and other (6%). Descriptive and demographic data for the program sample is outlined in Table 1.

From the 2012–2022 EHR data, there were 41,530 low-acuity ED visits for the 5,482 patients in the sample. This accounted for 26% of all low-acuity ED visits during this period, with an average of 3.27 low-acuity ED visits per patient in this sample. Overall, 3,693 (66.3%) patients had a lower ED visit rate after their program enrollment and 3,162 (58%) had at least 0.5 fewer visit per year. The mean number of low-acuity ED visits per patient was 1.73 pre- and 1.41 post-enrollment (*P*<0.001) across the entire study period. Furthermore, the mean expected ED visit rate post-enrollment was 2.5 visits per year and reduced by 1.12 visits per year (*P*<0.001) in observed rate, a 45% reduction.

Among patients “with a reduced low-acuity ED visit rate” after program enrollment, a smaller proportion were Black (94% vs 96% of those “without a reduced ED visit rate,” *P*<0.001) and used Medicaid (54% vs 64%, *P*<0.001), and a larger proportion had private insurance (18% vs 14%, *P*<0.001) or no insurance (14% vs 12%, *P*<0.001).

For acuity levels at index visits, patients with higher acuities (2 and 3), had a lower proportion “with a reduced low-acuity ED visit rate” (27%) compared to patients “without a reduced low-acuity ED visit rate” (65%). Conversely, patients with acuities 4 and 5 had greater proportions “with a reduced low-acuity ED visit rate” (73%) vs patients “without a reduced low-acuity ED visit rate” (35%) (*P*<0.001). None of the patients with the highest acuity of 1 (3) saw a reduced ED visit rate (Table 2). When analyzing proportions by excluding patients whose ED visit rate change was “near zero,” we found there was no longer a significant difference by race. All other proportions and significant differences remained the same across the same comparisons with no other notable changes.

For mean ED visit rate changes, uninsured patients had the highest reduction in average visit rate at 1.52 fewer visits per 12 months, post-enrollment. Medicaid and privately insured patients had similar mean reductions (1.08 and 1.15 fewer visits per 12 months, respectively). Lastly, Medicare patients had the lowest reduction in ED visit rate at 0.78 fewer visits per 12 months. Although interesting, these mean changes among payor groups were not statistically significantly different (*P* < .01). By acuity level at index visit, patients with low acuity (4 and 5) had a significant reduction in mean ED visits post-enrollment at 1.81 fewer per 12 months compared to higher acuity patients (2 and 3) at 0.074 fewer visits per 12 months (*P*<.001) (Table 3).

### Subanalyses

**Figure 1:**
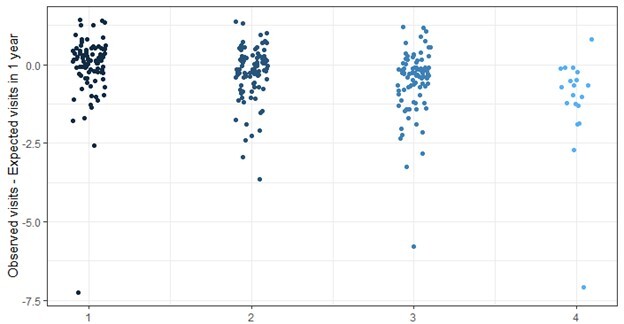
Change in low-acuity emergency department visit rate visits per 12 months for patients with multiple enrollments with a patient advocate from 1–4 or more (X-axis). Change in visit rate is calculated by observed visits post-program enrollment minus expected visits. *ED*, emergency department.

Of the 5,482 patients in the study sample, 537 (10%) were enrolled multiple times, often during a subsequent ED visit. Among the sample, 4,945 (90%) patients were enrolled once, 446 (8%) were enrolled twice, 72 (1%) three times, and 19 (<1%) were enrolled ≥4 times (maximum 11), totaling 6,137 program enrollments. When comparing those with “one enrollment” (4,945, 90%) vs “two or more’” enrollments (537, 10%), patient demographics were largely the same with the exception of two significantly different categories outlined in Table 4: payor status and acuity level. Patients with “one enrollment” compared with “two or more” had higher proportions of private insurance (18% vs 13%) and uninsured patients (13% vs 11%) and a lower proportion of patients with Medicaid (53% vs 70%). For acuity level at index visit, for those with “one enrollment” vs “two or more” proportions were lower for acuity level 2 (9% vs 14%), 3 (30% vs 36%) and 5 (4% vs 5%), whereas they had a higher proportion of acuity 4 (57% vs 45%). On average patients who enrolled multiple times had a reduction of 0.36 ED visit rate per year compared to a reduction of 0.15 for those with only one program enrollment. Difference between groups with one through four enrollments and their reduced ED visit rates are shown in Figure 1.

**Figure 2:**
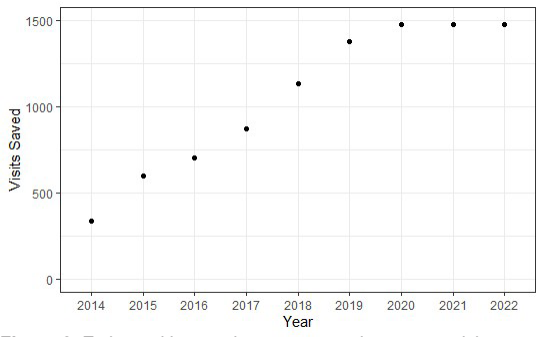
Estimated low-acuity emergency department visits prevented each year during the evaluation period. Points on the graph are inclusive of visits prevented.

The overall reduction in ED visit rates resulted in an estimated 9,447 fewer low-acuity ED visits. Averaged over the entire study period (2012–2022), the UCMC patient advocate intervention program resulted in 1,050 visits prevented per year. Furthermore, the average number of prevented visits increased each subsequent year until 2020, after which a consistent rate of 1,495 low-acuity ED visits prevented per year was observed from 2020–2022 (Figure 2).

### Healthcare Cost Analysis

The average direct variable cost for low-acuity patients referred to the MHSCC was $307 from July 2021–June 2022. When applied to the estimated 9,447 low-acuity ED visits prevented, this totaled $2,900,229 in healthcare expenditure avoided. Annually, this equated to an average of $322,247 per year. However, if applied to the stabilized rate of ED visits prevented at 1,495, this would prevent healthcare costs of $458,965 per year assuming maintenance of this prevention rate over time.

## DISCUSSION

This study identified a significant reduction in ED visits post-intervention among a subset of MHSCC program patients. This reduction was greater among patients with multiple program enrollments, and patients who had this reduction were more likely to be low-acuity at index visit and have private insurance or be uninsured. However, the overall intervention benefit by mean ED visits was only statistically different by acuity level. This continues the development of evidence on the effectiveness of like programs in an ED setting and among patients with attributes that are more likely effected by the program (payor group and acuity level).

The overall finding of reduced ED visits is not unique in the literature, yet it varies methodologically due to our use of a longer than standard time frame of seven years compared to the shorter outcome periods common in literature, such as 72 hours and 30 days for ED revisits and monitoring ED revisits for a median of six months post-intervention.^15^,^17^,^31^ Therefore, our findings also begin to demonstrate the longevity of overall program effectiveness by low-acuity ED visits prevented. Nevertheless, longevity of the program’s effectiveness was not directly analyzed in this study and requires further research. This longitudinal style design can also help account for unstudied confounding variables such as ED clustered visits by normalizing the data over a long period of time. Furthermore, we did not use a control group and/or propensity score matching (common in the literature) as there was a higher baseline of mean ED visits in the sample than the general UCMC ED population.^31^ This indicates some unknown factor or attributes of patients who were referred to this program by physicians, which is not accounted for by standard matching methods, potentially leading to selection bias.

Many similar program evaluations and research that do not develop control groups have had the overall quality of their studies called into question.^14^ In this case, for the development of a control group there were three different populations or samples available. This included the overall ED population; patients referred to the program who had not been assisted; and patients who refused assistance during attempted program enrollment. These presented various levels of selection bias. In the case of the general ED population, there was no clear index visit from which to calculate pre- and post-low-acuity ED visit rates. For patients who may not have been assisted (unable to reach, voicemail left, etc.) or had not been. reached out to at all, the MHSCC dataset had limitations for data on these patients and we could not delineate these two scenarios. For patients who refused assistance we realized that the overwhelming majority stated they already had an appointment set up/had a medical “home” or they wanted to call and schedule it themselves, indicating they did not need help getting established into a medical “home” and could represent a dedicated sample of patients who do not need help. Overall, this made it difficult to develop a matched control group from the general ED population.

The significant findings from this impact evaluation of the MHSCC reinforce program efforts toward reducing ED visit rates and highlights specific caveats and applications. This includes focusing on low-acuity patients and non-high ED users, although there is no agreed upon definition of high ED users.^17^ Patients with private insurance were more likely to benefit from this program; this finding could reflect a multitude of well-documented inequities in healthcare access and availability associated with patients on Medicaid and public insurance leading to reduced appointment adherence.^13^ Patients with Medicaid still had reduced ED visit rates, which has also been seen with prospective, randomized control trial research, and the mean reduction among Medicaid patients was similar to private payors.^26^ Although not significantly different, patients under the uninsured/self-pay group showed good response from the program, which aligns with research in a quasi-randomized trial.^32^ This is an important finding as many patients across the US lose health coverage if it is not provided by their employer, although further research needs to be done to examine patient advocate programs as avenues for securing reliable insurance for their patients.^33^ Other interesting findings include the accrual and stabilization of estimated low-acuity ED visits prevented per year over the course of this evaluation and magnitude of improvement from multiple program enrollments. It was expected that the ED prevention would fall off after enrollments had stopped in 2020 and during the two years afterward. However, this could have been influenced by COVID-19 pandemic responses and a national reduction in ED visits from 2020–2021.^4^,^7^–^9^ This phenomenon could have confounded the result by showing a greater ED visits prevention than would otherwise have been seen during these years.

When hypothesizing the drivers of the MHSCC’s impact, the broader context of the program’s institution must be considered. The South Side of Chicago has been experiencing higher disparities in healthcare access, ED usage (particularly for mental health emergencies), disability, poverty, unemployment, violent crime, food access, chronic disease mortality rates and other SDoH needs for the past few decades compared to the State of Illinois and the rest of the city of Chicago, potentially catalyzing the impact found.^28^ The MHSCC also connected to broader healthcare ecosystem initiatives, such as the Southside Health Collaborative and the Southside Healthy Community Organization, which have worked to establish a network of community-based healthcare clinics and hospitals to partner with University of Chicago Medicine to support patients across the South Side.^16^ Further evaluation on other outcome measures need to be investigated such as health literacy, appointment adherence or healthcare navigation knowledge; however, there is established literature on similar programs improving appointment adherence.^20^,^31^

The cost analysis done was comparable to former studies; however, several studies seen in review papers used fixed costs as a part of their healthcare cost-prevention estimates.^17^,^34^ We did not use this method as there was no known reduction in staff or units-closed event that would have reflected actual reduced spending on fixed costs such as physicians, staff or maintenance of a department or section of the hospital. Because the Urban Health Initiative is the main Community Benefit sector and oversite at UCMC, this is not a defined, expected or intended benefit of the program. The purpose of the MHSCC is to benefit patients and healthcare ecosystem on the South Side of Chicago. These findings are an unintended benefit indicating financial sustainability congruent with what similar programs have shown. That said, these referenced programs were focused on older populations and overall, there are uncertain conclusions throughout literature as there are limited in depth, statistical cost savings analyses.^17^,^22^,^35^,^36^

In continuing a cost-benefit analyses, there is an unintended threat to the ecosystem of healthcare on the South Side in that many patients seek to be treated at UCMC who are “out of network” (their health insurance plan is not contracted with the hospital), putting strain on both UCMC (did not accept their insurance policy) and safety net, community and federally qualified hospitals that are missing the reimbursement from treating these mostly Medicaid-insured patients. There is an unknown cost benefit and protections of patient billing by preventing patients from seeking care outside their network and helping them maintain quality care in institutions that are in their payor-group network.^37^

## LIMITATIONS

This study had several limitations. Because the program and study were conducted at a single-site our findings are not generalizable beyond the sample. Furthermore, data from other hospitals was not included, meaning it is unknown whether patients visited other EDs over the course of the study. However, UCMC is the most frequented ED on the South Side of Chicago by a significant margin for both high- and low-acuity patients, which could help lessen the effect of this limitation.^38^ The retrospective data used was for programmatic purposes and was not collected in a rigorously standardized way or complete manner throughout the study period. From 2018 onward, there was not a clearly defined or understood decision-making process for emergency physicians to refer patients to the program. Due to program staff turnover and lack of documentation, there was also limited knowledge on what guidelines or general information was provided to physicians during this time period. Although this created the foundation for a validated tracking system of program referrals, this was not incorporated into the MHSCC dataset. Currently the program uses emergency physician champions, quarterly ED resident physician conferences, and moderating clauses on referral orders in the EHR system for communication and tracks referrals to program outreach ratios.

The study also did not control for a wide range of covariates including COVID-19 ED visit reductions, Area Deprivation Index, chronic illness, mental illness, substance use disorder, specialty care follow-up services provided, and ED visit patterns including post-traumatic injury visits or other cluster visits, using the Poisson regression model. Additionally, UCMC opened their adult Level I trauma center in 2018, which changed the setting and potential patients approached for the program.

Although the MHSCC started in 2005, there was only program data available beginning in 2010. This means patients included in the study may have enrolled before the study period. Also, EHR data was only available from 2012 onward, limiting use of the program dataset to 2014–2020 so that there would be adequate time before the index visit to calculate pre-ED visit rates. Due to such a long period other considerations may have been missed such as accounting for census data (eg, urban, county or state migration).

## CONCLUSION

Emergency department use in the United States is still an immense healthcare burden with many visits being low acuity or treatable outside an ED.^1^,^2^,^11^ This evaluation study found a significant reduction in patients’ low-acuity ED visit rate for Medical Home and Specialty Care Connection Program participants and provides evidence that patient advocate and navigation programs can be effective and sustainable for providing services to ED patients. The MHSCC warrants further evaluation on the outcomes of the program, the mechanisms of physician referrals, and attributes of the patient population to understand what specifically drives success of this intervention.

## Figures and Tables

**Table 1:** Demographic breakdown and proportions of evaluation sample size: patients who engaged with a patient advocate.

Patient Descriptive Categories		Total Patients
N		5,482
Sex		
	Male	2,070 (38)
	Female	3,412 (62)
Race		
	Black	5,183 (95)
	Not Black	299 (5)
Ethnicity		
	Hispanic or Latino	126 (2)
	Non-Hispanic or Latino	5,356 (98)
Age		
	16 –18	158 (3)
	19 – 33	2,487 (45)
	34–48	1,353 (25)
	49 – 64	1,080 (20)
	65 – 78	306 (6)
	79 +	98 (2)
	Mean	38.4
Insurance/Payor Status		
	Private	936 (17)
	Medicaid	3,322 (61)
	Medicare	507 (9)
	Uninsured/Self-Pay	717 (13)
Acuity Level of Patients during Index Visit		
	1	3 (<1)
	2	513 (9)
	3	1,658 (30)
	4	3,076 (56)
	5	225 (4)

**Table 2:** Demographic and frequency statistic comparisons of patients who had a reduced low-acuity emergency department visit rate to those who did not. This includes all patients with either an ED visit rate change of less than 0 (had reduced visit rate) or greater than 0 (did not have a reduced visit rate).

Patient Descriptive Categories	Patients with Reduced Low-Acuity ED Visit Rate, Post Enrollment	Patients without a Reduced Low Acuity ED Visit Rate, Post Enrollment	P-values
N	3,639	1,843	
Sex			
Male	1,409 (39)	661 (36)	.04
Female	2,230 (61)	1,182 (64)	
Race			
Black	3,414 (94)	1,769 (96)	<.001*
Not Black	225 (6)	74 (4)	
Ethnicity			
Hispanic or Latino	93 (3)	33 (2)	.07
Non-Hispanic or Latino	3,546 (97)	1810 (98)	
Age			
16 –18	106 (3)	52 (3)	.71
19 – 33	1,646 (45)	841 (46)	
34 – 48	903 (25)	450 (24)	
49 – 64	711 (20)	369 (20)	
65 – 78	213 (6)	93 (5)	
79 +	60 (2)	38 (2)	
Mean Age	38.5	38.5	-
Insurance/Payor Status			
Private	673 (18)	263 (14)	<.001*
Medicaid	2,134 (59)	1,188 (64)	
Medicare	332 (9)	175 (9)	
Uninsured/Self-Pay	500 (14)	217 (12)	
Acuity Levels of Patients during Index Visit			
High acuity (2 and 3)	982 (27)	1,189 (65)	<.001*
Low acuity (4 and 5)	2,654 (73)	647 (35)	

Significant findings (*) are for *P*-values of < 0.001.

*ED*, emergency department.

**Table 3:** Summary of mean change in ED visit rates per 12 months for descriptive categories from Table 2 with statistical significance between patients with a reduced ED visit rate and those without (excluding Race).

Mean Rate of Change for ED Visits, Post-Program Enrollment (per 12 Months)			P-values

Patient Descriptive Categories		Mean Change Visits (per 12 Months)	
N	5,482		
Payor Status			
	Medicaid	−1.08	<.01
	Private	−1.15	
	Medicare	−0.78	
	Uninsured	−1.52	
Acuity			
	Low Acuity (4 and 5)	−1.81	<.0001**
	High Acuity (2 and 3)	−0.074	

Significant findings (*) are for p-values of < 0.001 and (**) for <0.0001.

**Table 4:** Demographic and frequency statistic comparisons between patients who had one program enrollment and those who had two or more.

Descriptive Category		Patients with one program enrollment	Patients with two or more program enrollments	*P*-values
N		4,945	537	
Sex				
	Male	1,880 (38)	190 (35)	.21
	Female	3,065 (62)	347 (65)	
Race				
	Black	4,661 (94)	522 (97)	<.01
	Not Black	284 (6)	15 (3)	
Ethnicity				
	Hispanic or Latino	122 (2)	4 (1)	.02
	Non-Hispanic or Latino	4,823 (98)	533 (99)	
Age				
	16 –18	145 (3)	13 (2)	.03
	19 – 33	2,276 (46)	211 (39)	
	34–48	1,213 (25)	140 (26)	
	49 – 64	951 (19)	129 (24)	
	65 – 78	275 (6)	31 (6)	
	79 +	85 (2)	13 (2)	
	Mean Age	40.3	40.7	
Insurance/Payor status				
	Private	882 (18)	54 (10)	<.001*
	Medicaid	2,624 (53)	378 (70)	
	Medicare	460 (9)	47 (9)	
	Uninsured/Self-Pay	659 (13)	58 (11)	
Acuity level of patients during index visit				
	1	2 (<1)	1 (<1)	<.001*
	2	439 (9)	74 (14)	
	3	1,465 (30)	193 (36)	
	4	2,836 (57)	240 (45)	
	5	196 (4)	29 (5)	

* Indicates statistically significant comparisons with *P* < .001.
